# PMMA-Based Nanocomposites for Odontology Applications: A State-of-the-Art

**DOI:** 10.3390/ijms231810288

**Published:** 2022-09-07

**Authors:** Ana M. Díez-Pascual

**Affiliations:** Universidad de Alcalá, Facultad de Ciencias, Departamento de Química Analítica, Química Física e Ingeniería Química, Ctra. Madrid-Barcelona Km. 33.6, 28805 Alcalá de Henares, Madrid, Spain; am.diez@uah.es

**Keywords:** PMMA, polymer nanocomposites, dentistry, nanoparticles, biomaterials

## Abstract

Polymethyl methacrylate (PMMA), a well-known polymer of the methacrylate family, is extensively used in biomedicine, particularly in odontological applications including artificial teeth, dentures and denture bases, obturators, provisional or permanent crowns, and so forth. The exceptional PMMA properties, including aesthetics, inexpensiveness, simple manipulation, low density, and adjustable mechanical properties, make it a perfect candidate in the field of dentistry. However, it presents some deficiencies, including weakness regarding hydrolytic degradation, poor fracture toughness, and a lack of antibacterial activity. To further enhance its properties and solve these drawbacks, different approaches can be performed, including the incorporation of nanofillers. In this regard, different types of metallic nanoparticles, metal oxide nanofillers, and carbon-based nanomaterials have been recently integrated into PMMA matrices with the aim to reduce water absorption and improve their performance, namely their thermal and flexural properties. In this review, recent studies regarding the development of PMMA-based nanocomposites for odontology applications are summarized and future perspectives are highlighted.

## 1. Introduction

Polymethyl methacrylate (PMMA) is the most well-known polymer of the methacrylate family, obtained from the in-chain polymerization of methyl methacrylate. It is a low-density, stable, and durable polymer that presents excellent biocompatibility and hemocompatibility, and high transparency, making it suitable for a wide variety of biomedical applications that require lasting, mechanically stable structures such as orthopedics and bone tissue engineering [1,2]. Furthermore, its aesthetics, inexpensiveness, processability, versatility in terms of shaping, simple manipulation, low density, and adjustable mechanical properties make it a perfect candidate in the field of dentistry, with applications such as the manufacture of artificial teeth, interim-fixed restorations, dentures and denture bases, obturators, provisional or permanent crowns, and occlusal splints [3,4]. Nonetheless, PMMA lacks antimicrobial properties [5], shows high water absorption, and displays poor flexural and impact strength [6], which restricts its use in certain applications. With the aim to solve these issues and expand its range of uses, different approaches have been carried out including the development of PMMA composites. In this regard, composites comprising natural or synthetic fibers [7,8], natural particles [9], metallic nanoparticles [10], metal oxide nanofillers [11,12], carbon-based nanofillers [13], and more have been developed. Filler nature, shape, size, concentration, and distribution play a key role in the performance of the resulting composites [14]. Furthermore, the interactions between the particles and the matrix also influence the properties, and these can be tailored via particle surface treatment, which enhances the interfacial adhesion between the composite components.

Despite PMMA has been used in dental applications for many years, strong efforts have recently focused on improving its properties. In this review, current studies dealing with PMMA nanocomposites incorporating different types of organic and inorganic nanomaterials for odontological applications will be summarized, and future perspectives in the field will be suggested.

## 2. PMMA: Properties and Applications

Poly [1-(methoxy carbonyl)-1-methyl ethylene])], PMMA, is typically synthesized via free radical addition and polymerization of methyl methacrylate (C_5_O_2_H_8_) to form poly methylmethacrylate (C_5_O_2_H_8_)_n_ [15]. The polymerization starts via free radical formation, either chemically or with energy such as heat, light, or microwaves. During the propagation, polymerization continues through the binding of monomers, and finally, the polymerization ends via transfer of free electrons to the chain edge.

The properties of PMMA-based materials depend on the source used for the polymerization and curing. Properly cured PMMA materials have good biocompatibility and physical properties for odontological applications, including low density (1.18 g/cm^3^), high light transmission (92% of visible light), and transparent color. They are amorphous materials with a glass transition temperature (T_g_) in the range of 95–125 °C. However, the thermal conductivity of PMMA is relatively low (5.7 × 10^−4^ °C·cm^−1^) [16], and this is a drawback since denture base materials should have adequate thermal conductivity to dissipate the food temperature to the oral tissues of the patient. If the heat is transmitted slowly, it can result in surface cracking. Additionally, the low conductivity can influence the ability to sense food temperature compared to metallic denture bases, which are highly conductive. Moreover, it presents a high coefficient of thermal expansion (81 × 10^−6^/°C [16]).

On the other hand, denture base materials should have good color stability and not lose color over time [17]. Nevertheless, PMMA-based materials typically show poor color stability due to the release of residual monomers that promote water absorption and consequently, discoloration. Other factors including porosity generated during the manufacturing process and regular consumption of beverages such as coffee, tea, alcohol, etc. can lead to discoloration; hence, PMMA dentures may need replacement after some time.

Another issue is the water absorption, which takes place when materials are immersed. Due to PMMA polarity, the water molecules penetrate the polymer chains and act as plasticizers [18]. The infiltrated water molecules can produce an expansion of PMMA and affect its dimensional stability. Hence, the water absorption (like the solubility) should be minimal. The absorption is typically about 0.7 mg/cm^2^, which, consequently, meets the requirements of the ISO 20795-1 [19].

Another important property is the polymerization shrinkage, which may result in noteworthy dimensional changes and imprecisions during denture fabrication [20]. Therefore, low levels of polymerization shrinkage are desired for these applications. In general, PMMA-based materials show a curing shrinkage in the range of −0.50 to −0.58%. Certain fillers, such as carbon nanotubes [21], can significantly reduce the polymerization shrinkage. Radiopacity is another physical property desired in dental materials. However, due to its polymeric nature, PMMA is a radiolucent material, and it is difficult to detect in radiographs [22]. Inducing radiopacity via modification is still a challenge given that salts of heavy metals are incompatible with PMMA.

Regarding mechanical performance, it is important to note that odontological base materials are subjected to complex masticatory stresses in the oral cavity. Consequently, good mechanical properties are required. In particular, a high flexural strength is required to tolerate the mastication forces without permanent deformation or fracture [4]. The flexural strength of PMMA is in the order of 90 MPa. However, it can be affected by several factors, including the curing process, degree of polymerization, storage conditions, etc. The fracture toughness of PMMA, which indicates its ability to resist crack propagation, is about 2 MN/m^3/2^, and the impact strength is relatively low, at around 1J. High impact strength is desired to avoid fracture when subjected to a high impact force, such as unintentional fall. The wear resistance of PMMA is low compared to molding alloys and dental porcelains [23]. The wear resistance is directly related to the surface hardness, which is about 300 MPa in neat PMMA and lower than alloys and porcelains [24].

**Figure 1:**
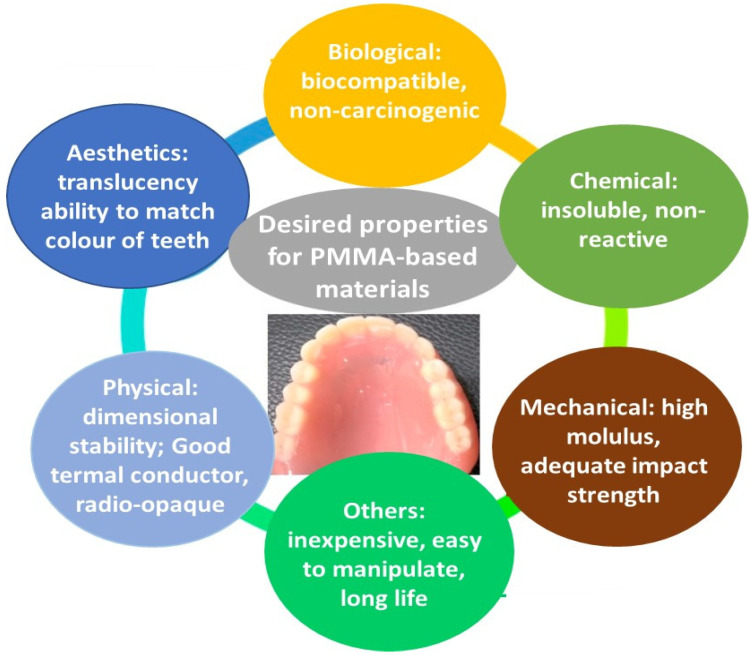
Ideal properties of PMMA-based materials for odontological applications.

Dentistry base materials should be inert and chemically non-reactive with oral fluids and nutrients. PMMA materials are organic resins that have low solubility (0.02 mg/cm^2^ in water and 0.04 mg/cm^2^ in hydrocarbons). However, their solubility in ketones and esters is relatively high. Besides, alcohols act as plasticizers and may decrease the T_g_. Therefore, storage or cleaning of dentures with alcohols should be skipped. Taking into account the aforementioned data, it is clear that PMMA fulfills many requirements. However, several drawbacks, including the detrimental thermal properties (low thermal conductivity or diffusivity, high coefficient of thermal expansion), mechanical properties (brittleness, low hardness and wear resistance, poor fatigue strength), discoloration, susceptibility to warpage, and porosity, still require improvement and further investigations. A scheme of the ideal properties of PMMA-based materials for odontological use is depicted in Figure 1.

PMMA materials are extensively used for a range of applications, including engineering, healthcare, and dentistry. In addition to denture bases, other oral healthcare applications are artificial teeth, impression trays, temporary crowns and bridges, obturators for cleft palates, occlusal splints, denture relining, and repair, and so forth.

## 3. Nanofillers Used in PMMA Nanocomposites

**Figure 2:**
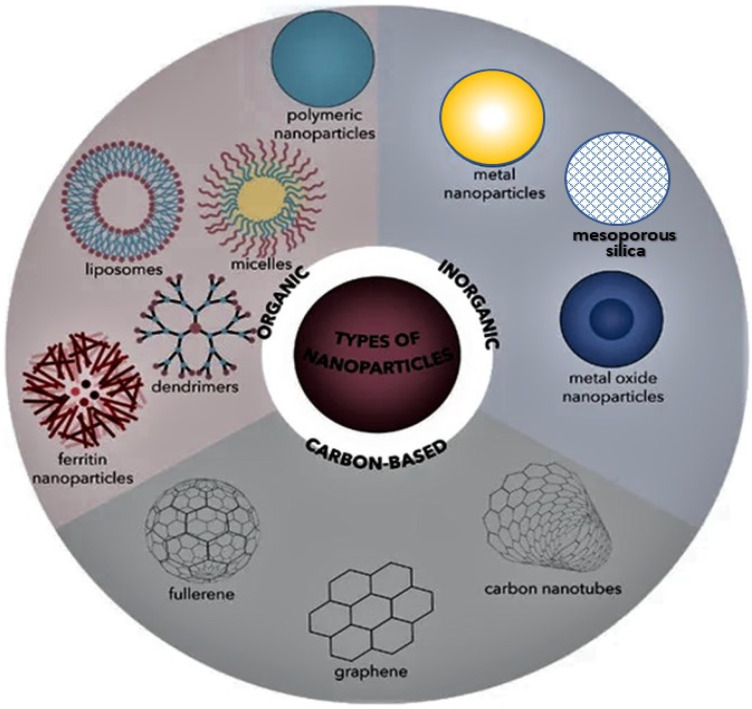
Different types of nanomaterials used as nanofillers in polymeric nanocomposites, categorized according to their nature into inorganic, organic and carbon-based.

Different types of nanomaterials have been used to improve the properties of polymeric matrices. According to their nature, these nanofillers can be classified into three main groups, as depicted in Figure 2: (1) organic, including dendrimers, micelles, liposomes, polymer nanoparticles (NPs), and ferritin; (2) inorganic, including metal NPs (Ag, Au, Cu), metal oxide NPs (e.g., Fe_3_O_4_, ZnO, MgO, TiO_2_), hydroxyapatite (HA), and mesoporous silica; (3) carbon-based, including fullerenes, quantum dots, carbon nanotubes, graphene, and nanodiamond [25,26]. In the following section, those typically used as nanofillers in PMMA matrices will be briefly described.

### 3.1. Metallic Nanoparticles

AgNPs are widely studied among metallic NPs since they have a huge range of applications in arenas, ranging from medicine and pharmacology to food technology, water purification, etc. [27,28]. They can be synthesized via numerous approaches including sol–gel, hydrothermal, thermal decomposition, CVD, microwave-assisted combustion, and so forth [28,29]. They are strong antibacterial [30] and antifungal agents [31] as well as an anticancer tool [29] due to their plasmonic features. Their specific mechanism of toxicity is still unclear, though it has been proposed that it is related to their ability to liberate silver ions (Ag^+^) [30], stimulating molecular pathways that provoke cell death. In particular, in the field of dental medicine, the use of AgNPs in different kinds of dental prosthesis matrices could be a fundamental tool in immunodepressed patients that suffer from different oral infections. For instance, they show a strong inhibition towards *C. albicans* [32], a pathogenic yeast with a high colonization ability that provokes oral cavity infection due to its ability to develop biofilms on different materials. AgNPs antifungal action is higher than fluconazole, the drug usually used against the yeast. These antibacterial and antifungal properties account for their use in the biomedical field, and, in particular, in dental prostheses, where AgNPs could reduce the oropharyngeal candidiasis.

PtNPs are low-allergy and non-genotoxic nanomaterials for the organism, and they are of great interest due to their highly catalytic activity. They are currently being evaluated for their ability to reduce inflammation and they have also been applied in the biomedical field [33]. The antibacterial activity of Pt was first reported by Rosenberg et al. [34], who demonstrated its inhibitory activity on *Escherichia coli.* The main mechanism of activity seems to arise from the contact between PtNPs and bacteria, which promotes chemical interactions that disintegrate the bacterial cell. Furthermore, these NPs can scavenge reactive oxygen species (ROS), and free radicals from antioxidant responses can induce chain reactions that harm the bacteria.

### 3.2. Metal Oxide Nanoparticles and Ceramic Nanofillers

TiO_2_ (titania) is an FDA-approved compound for food, drugs, cosmetics, and food packaging uses. It exists in three main polymorphs, namely, anatase, rutile, and brookite [35]. It shows outstanding mechanical properties, high thermal conductivity, strong UV absorption, high hydrophilicity, and very good antibacterial activity against viruses, fungi, and bacteria [36]. It has been widely used for the development of antimicrobial coatings, which can be applied in the biomedical and odontological field [37,38]. Synthetic routes for TiO_2_ NPs include the sol–gel, hydrothermal and solvothermal methods, precipitation, and electrochemical processes, using titanium chloride, titanium isopropoxide, or titanyl sulfate-based compounds as precursors. Nevertheless, these techniques are disadvantageous in terms of reaction time and particle size control; hence, new green and inexpensive synthesis methods are being investigated.

ZrO_2_ (zirconia) is a ceramic material with excellent erosion, corrosion, and abrasion resistance along with excellent fracture toughness as well as compressive and flexural strength. Its fine grain size enables excellent surface finishes and the ability to hold a sharp edge [39]. Recently, many investigations have focused on adding modified ZrO_2_ nanoparticles such as yttria-stabilized tetragonal zirconia polycrystals (Y-TZP) to improve the mechanical and physical properties of conventional heat-polymerized denture base resins [40]. This type of zirconia, called “ceramic steel”, possesses superior mechanical properties, good surface properties, and high biocompatibility, thus making it an attractive option for many dental applications.

Mesoporous SiO_2_ (silica) particles, with a pore size between 2 and 50 nm, are promising for biomedical applications due to their excellent biocompatibility, water dispersibility, low toxicity, thermal stability, and easy and large-scale synthesis. Their particle size, pore size, crystallinity, level of porosity, and shape can be finely tuned by modifying the synthesis parameters, making them suitable for specific applications [41]. They are typically synthesized from a silica source (i.e., tetraethyl orthosilicate, TEOS), which is mixed with a surfactant and then a hydroxide is added, allowing the hydrolysis of the silicate. The surfactant can then be removed via calcination, solvent extraction, or dialysis. Frequently, a co-surfactant is added to attain nanoparticles with controlled size [42]. They can also be synthesized via a hydrothermal treatment or a sol−gel process which uses organo-silane precursors that generate the sol via hydrolysis and condensation. Also, many surface modifications of these mesoporous NPs have been reported, such as the addition of polyethylene glycol (PEG) or silane treatments that enable them to incorporate amino or carboxylic groups, and hence, to control the surface chemistry for the desired purposes.

Al_2_O_3_ (alumina) nanoparticles are porous nanomaterials with a corundum-like structure, and they are also biocompatible, inexpensive, and easy to handle; additionally, they show a very high surface area, high thermal stability, low electrical conductivity, and excellent biomechanical and tribological properties such as resistance to wear and abrasive environments, chemicals, and mechanical stresses [43]. In addition, their bioinertness and easy surface functionalization allows their use in the biomedical field. They can be synthesized using simple and cost-effective approaches such as mechanical ball milling, laser ablation, solution reduction, gas decomposition, and so forth. They are also well known for their antimicrobial effects [44,45]. The antimicrobial properties against *E. coli*, *B. subtilis*, *P. fluorescens*, and *S. Epidermis* have been demonstrated, ascribed to the electrostatic interactions between the NPs and the bacterial cells [45].

Calcium phosphate bioceramics such as hydroxyapatite (HA) also show extraordinary properties for biomedical applications including biodegradation, osteoconduction, osteointegration, biocompatibility, and composition and structure similar to natural bone and teeth [46]. Moreover, the stiffness, density, and bioactivity of HA, its ability to chemically bond to bone and lack of toxicity make it a preferred reinforcement as a bone cement [47]. In addition, it has been used in restorative and preventive dentistry, in periodontology, and in oral and maxillofacial surgery [48,49].

### 3.3. Carbon-Based Nanofillers

Diverse allotropes from carbon have been used as nanofillers in polymeric nanocomposites, including nanodiamond (ND), carbon nanotubes (CNTs), graphene (G), and its derivatives, graphene oxide (GO) and reduced graphene oxide (rGO). CNTs were initially reported in 1991 [50], and consist of 1D, cylindrical layers of carbon atoms. They can be categorized into single-walled carbon nanotubes (SWCNTs), with only one carbon layer rolled up forming a cylinder; double-walled carbon nanotubes (DWCNTs), with two concentric carbon layers; or multi-walled carbon nanotubes (MWCNTs), with several concentric carbon layers. They are lightweight, and have outstanding mechanical, thermal, and electrical properties, which depend on their diameter, length, and chirality [51]. Their stiffness is the highest amongst any identified material, with an elastic modulus of about 1000 GPa and a tensile strength of about 35 GPa [52]. They show very high electrical conductivity (higher than metals like Ag), very high thermal conductivity (more than 10^3^-fold that of metals such as Cu), and display very high thermal stability, up to 800 °C in an oxidative environment and 2500 °C under vacuum [53]. However, they have a great predisposition to aggregate and form ropes, which leads to a worsening of certain properties, particularly mechanical and electrical. Henceforth, functionalization with polymers [54,55] or other molecules is frequently required. The most common methods to synthesize CNTs are chemical vapor deposition (CVD), electric arc discharge, and laser ablation [54]. CVD is a technique in which the vaporized reactants (hydrocarbon gases) react chemically inside a quartz tube filled with inert gas, which is placed in a furnace kept at high temperatures (500–900 °C). The hydrocarbon gases are pumped into the quartz tube, undergo pyrolysis reaction, and form vapor carbon atoms that deposit onto a substrate with metal catalyst nanoparticles of Fe, Co, and Ni. In the arc discharge, a potential is applied across pure graphite electrodes maintained at a high pressure of inert gas filled inside a quartz chamber. When the electrodes strike each other, an electric arc is generated, and the energy is transferred to the anode, which ionizes the carbon atoms of pure graphite and produces C^+^ ions in the form of plasma. These positively charged ions move towards the cathode, where they are reduced, deposited, and grown as CNTs [46]. The laser ablation method is a physical vapor deposition method in which a graphite target placed in a quartz chamber filled with inert gas is vaporized by a laser source. The vaporized target atoms are swept toward a cooled copper collector by the flow of inert gas, where they are deposited and grown.

Nanodiamonds (NDs) have excellent mechanical and thermal properties, high surface area, and tunable structures. They have high strength, chemical stability, high thermal conductivity, bioinertness, and are biocompatible, non-toxic, and suitable for biomedical applications, including fixed interim prostheses [56]. They are inexpensive and have great potential for surface functionalization. They can be naturally produced by an explosion or meteoritic impacts and obtained in the laboratory via hydrothermal synthesis, laser bombardment, chemical vapor deposition, and electrochemical synthesis [57].

Graphene (G) is a 2D, atomically thick carbon nanomaterial comprised of a honeycomb lattice of sp^2^ carbon atoms [58]. It has outstanding electrical, optical, and thermal properties, combined with high mechanical resistance, transparency, low density, and flexibility. For instance, it has a thermal conductivity in the range of 3000–5000 W m^−1^ K^−1^ [59], about 10-fold higher than that of other metals like Cu, very high electron mobility (20,000 cm^2^ V^−1^ s^−1^), and exceptional electrical conductivity (up to 5000 S cm^−1^). Besides, it is one the strongest materials on earth, with a Young’s modulus of around 1 TPa and a tensile strength of about 120 GPa, which is significantly stiffer than steel [60]. These exceptional properties make G a perfect candidate for many applications such as sensors, supercapacitors, flexible electronic devices, and even biomedicine [61,62].

G synthesis is typically performed two ways, namely through bottom-up and top-down approaches [63]. In the top-down methods, the initial material is graphite, which can be exfoliated mechanically (scotch tape method), in the liquid phase (typically with the aid of ultrasounds to disperse the graphene layers) or electrochemically, which is based on the penetration of graphite by ions from the electrochemical solution using a potential [64,65].

On the other hand, G derivatives are currently used for numerous applications, including the fabrication of biosensors or dental resins to prevent microbial adhesion [66]. Amongst them, the most important is graphene oxide (GO), an oxidized form of G with oxygenated functional groups, mainly carboxylic groups on the edges and epoxy and hydroxyl groups on the layer plane, typically synthesized via Hummer’s method using strong oxidizing agents such as sulfuric or nitric acid [67,68]. Another well-known derivative is reduced graphene oxide (rGO), which is obtained via the thermal treatment of GO to remove functional groups or the chemical reduction of GO using synthetic reducing agents such as hydrazine or sodium borohydride, or, more recently, eco-friendly, natural reducing agents such as amino acids (i.e., ascorbic acid) or plant extracts.

## 4. PMMA-Based Nanocomposites for Odontological Uses

With the aim of improving PMMA properties, a variety of nanoparticles have been investigated, including metallic, ceramics, carbon nanotubes, and so forth. The studies performed demonstrated that the addition of nanoparticles did not lead to biocompatibility issues and enhanced several PMMA properties, namely mechanical and tribological [9,10,11,12,69,70,71,72,73,74,75,76,77,78], thermal [10,79,80,81,82], and antimicrobial [83,84,85,86,87,88,89,90,91,92] properties. They also decreased water absorption and solubility [93,94]. The improved properties of the nanocomposites arise from their characteristic features, such as a very large surface area and uniform distribution. In the following sections, specific examples of each type of nanofiller will be briefly discussed.

### 4.1. PMMA Nanocomposites with Metallic Nanoparticles

**Figure 3:**
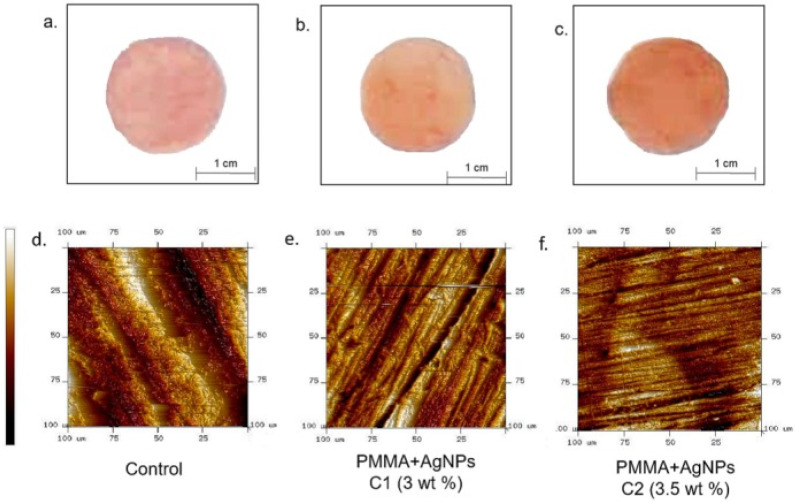
Images of PMMA without NPs (control) (**a**); PMMA + AgNPs (3 wt%) (**b**) and PMMA + AgNPs (3.5 wt%) (**c**): The color became darker with increasing concentrations of the NPs. AFM topographical images of PMMA without NPs added (control) (**d**); PMMA + AgNPs (3 wt%) (**e**) and PMMA + AgNPs (3.5 wt%) (**f**). Reprinted from ref. [95], copyright 2019, MDPI.

Amongst the most widely used NPs for dental applications are AgNPs owed to their strong antimicrobial activity even at low concentrations, as mentioned earlier. In particular, in the field of dental medicine, the use of AgNPs in different types of dental prostheses could be an essential tool in patients that suffer from oral infections. De Matteis et al. [95] added monodispersed citrate-capped AgNPs (20 nm size) at concentrations of 3 and 3.5 wt% in PMMA. Topographical evaluation of the nanocomposites via atomic force microscopy (AFM) showed a noticeable reduction in surface roughness (Figure 3). The *C. albicans* colonization on PMMA surfaces was assessed via the Miles and Misra technique as well as via scanning electron microscopy (SEM) at 24 and 48 h, which revealed the drop in yeast viability upon contact with the AgNPs. Acosta-Torres et al. [86] developed biocompatible and antifungal PMMA/AgNP nanocomposites. Results revealed that no damaging effect on cellular viability and cell proliferation nor genotoxic harm to mouse embryonic fibroblasts were produced. Furthermore, the nanocomposites were found to strongly reduce the adhesion of *C. albicans*. de Souza Neto and coworkers [96] incorporated several contents of AgNPs in PMMA to reduce denture stomatitis caused by *C. glabrata*. The nanocomposite with 0.05 wt% NPs had a strong ability to inhibit the biofilm formed on its surface, as demonstrated by the microbiological adhesion test against this fungi. The antimicrobial activity did not grow linearly with the nanoparticle content and was influenced by the state of dispersion of the NPs within the PMMA matrix. Also, a high AgNP concentration led to a decrease in the flexural strength, although some other mechanical properties were improved.

Other studies showed that AgNPs improved the compressive strength and thermal conductivity of PMMA [10] since the metallic nature of these NPs allows very high thermal conduction. The addition of AgNPs and graphene to PMMA significantly enhanced the mechanical properties (tensile, compressive, and flexural strengths) and lowered the water absorption [94]. Conversely, another work indicated that these NPs hardly changed the flexural strength [81]. Overall, it seems that the most important improvement upon AgNP addition is the antimicrobial and antifungal activity, which has been thoroughly investigated.

**Figure 4:**
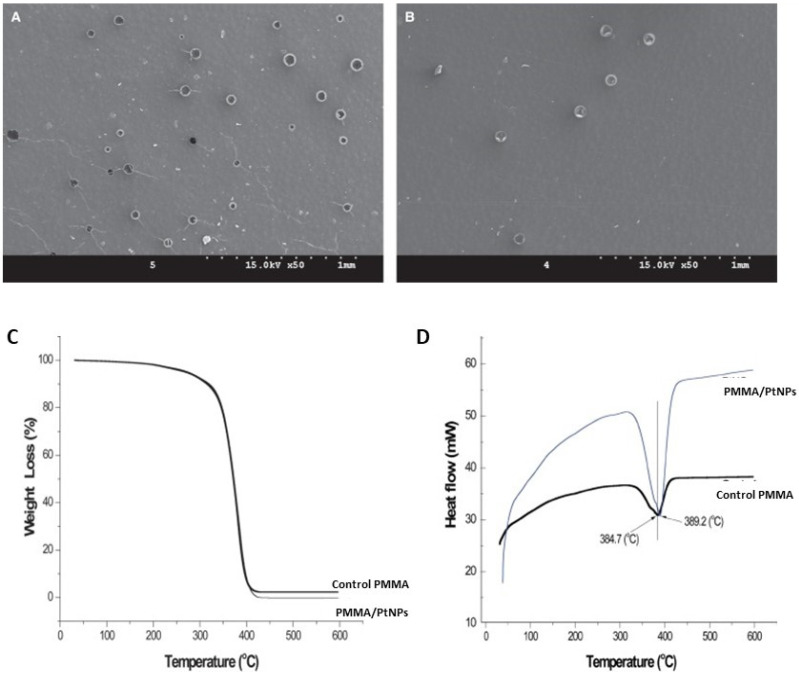
SEM image of control PMMA (**A**) and PMMA/PtNP nanocomposite (200 mg/L) (**B**). TGA curves of PMMA and PMMA/PtNP (**C**), showing overlapping curves in the range 350–400 ℃. DSC thermograms of PMMA and PMMA/PtNP (**D**), ranging from 30 ℃ to 600 ℃, the melting point of the nanocomposite (389.2 ℃) is slightly higher than that of the control (384.7 ℃). Reprinted from ref. [96], copyright 2019, Elsevier.

PtNPs were also incorporated into PMMA to develop antimicrobial nanocomposites [97], which were tested against *S. mutans* and *S. sobrinus*. The PMMA/PtNP nanocomposites showed a bacterial anti-adherent impact rather than a bactericidal effect above a concentration of 50 mg/L when compared to bare PMMA with very small amounts of Pt^+^ eluted, along with superior mechanical properties. According to SEM analysis, they exhibited similar surface texture with slightly greater surface cracking and blistering than pure PMMA (Figure 4a,b). Furthermore, according to TGA and DSC analyses, the nanocomposites had comparable thermal stability and a somewhat higher melting point than the neat matrix (Figure 4c,d, respectively), demonstrating improved thermal properties. For dental applications, the long-term effect of this activity, biocompatibility, and color stability should be investigated. In another study, it was found that each metallic NP can have a different effect on the PMMA matrix: while Ag and Pt can improve bending deflection and Pd can enhance bending strength and Vickers hardness [98].

Other nanoparticles such as AuNPs have also been included in PMMA, and results revealed that the nanocomposites had improved thermal conductivity and viscoelastic response compared to the neat resin [14].

### 4.2. PMMA Nanocomposites with Metal Oxides and Bioceramics

**Figure 5:**
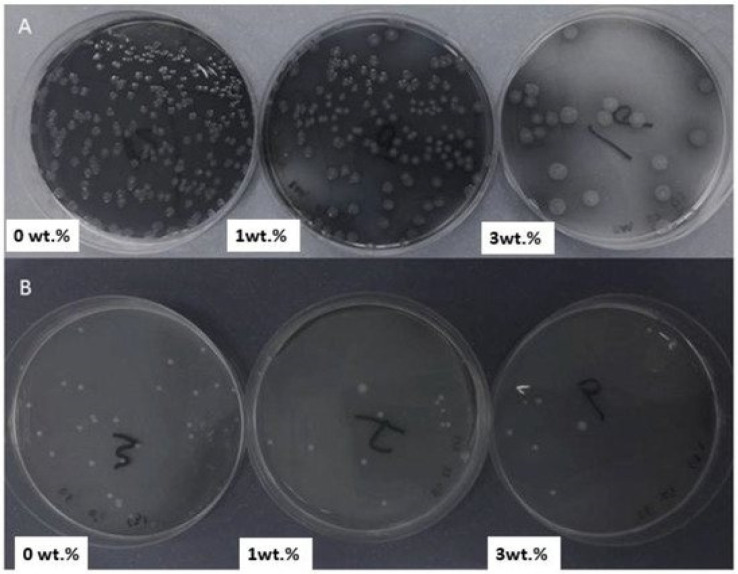
C.F.U. of *P. aeruginosa* (**A**) and *E. faecalis* (**B**) on nutrient agar after growing it for 48 h on PMMA composites containing 0, 1, and 3 wt% TiO_2_. Reprinted from ref. [100], copyright 2018, MDPI.

Numerous ceramic nanoparticles have been recently used to reinforce PMMA resin for odontological uses. These nanofillers can aid in the development of inexpensive nanocomposites with good mechanical performance and antimicrobial behavior. In particular, the impact strength, flexural strength, and fracture toughness of the base materials can be improved by modifying the nanofiller surface with silane coupling agents [99]. Alamgir et al. [78] developed PMMA/TiO_2_ nanocomposites with different nanofiller percentages via melt blending, and a uniform TiO_2_ dispersion in the polymer matrix was observed by SEM, which was reflected in the enhanced mechanical properties, as confirmed by micro-indentation and scratch tests. The presence of TiO_2_ improved the hardness, modulus, creep-recovery, and relaxation behaviors of these nanocomposites. The antimicrobial activity of PMMA was also enhanced with TiO_2_ due to decreasing bacterial adherence. Thus, Alrahlah et al. [100] found reduced colony-forming units of *P. aeruginosa* and *E. faecalis* on nutrient agar after growing them for 48 h on the nanocomposites compared to control PMMA, and the antibacterial activity improved with increasing NP content, with the optimum for the nanocomposite being 3 wt% TiO_2_ (Figure 5). On the other hand, the best nanoparticle dispersion and the highest increment in tensile strength were found for the nanocomposite with 1wt% TiO_2_ NPs. Higher NP loading led to reduced strength due to particle agglomeration.

In another study [101], two types of PMMA were reinforced with 1 and 5 wt% TiO_2,_ and it was found that the flexural strength and microhardness noticeably decreased and increased, respectively, with increasing TiO_2_ loading. Furthermore, TiO_2_ has been coated with fluorite and apatite and incorporated into PMMA, leading to improved antifungal effect against *Candida* growth [88].

**Figure 6:**
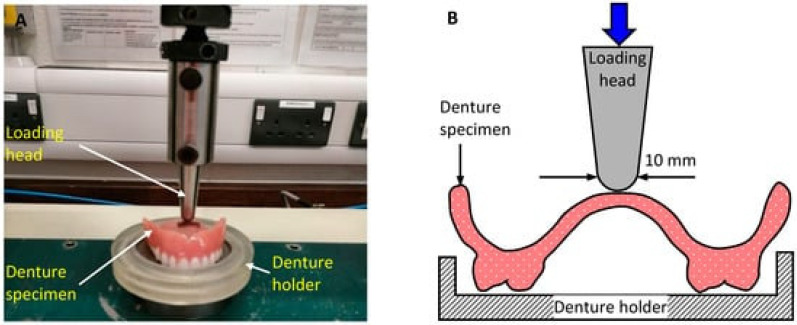
(**A**) Application of bending load on the palatal surface of PMMA/ZrO_2_ denture specimen using a Hounsfield universal testing machine and (**B**) schematic diagram of loading conditions. Reprinted from ref. [12], copyright 2020, MDPI.

Another promising NP to enhance the performance of PMMA are ZrO_2_. Gad and coworkers [72] investigated the effect of these NPs on the translucency and tensile strength of PMMA for removable prostheses. Upon increasing ZrO_2_ concentration, the strength gradually increased while the translucency was reduced. Significant improvements in mechanical properties, including fracture toughness, compressive and fatigue strengths, as well as superior thermal conductivity have also been reported in other studies [11,12]. Thus, Zidan and coworkers [12] investigated the flexural strength and fatigue of high-impact heat-polymerized PMMA resin reinforced with 3 and 5 wt% ZrO_2_ molded into denture specimens. Fatigue bending load was applied on the palatal surface of the denture specimen using a mastication simulator (Hounsfield universal testing machine), and equivalent flexural strength was calculated with data from bending tests with and without fatigue cyclic loading (Figure 6). Also, the fractured surface of the samples was examined by SEM. Bending tests demonstrated that the flexural strength of PMMA with 5 wt% ZrO_2_ increased significantly, by about 27%, compared to neat PMMA without any fatigue loading. However, the application of fatigue cyclic loading hardly enhanced the equivalent flexural strength of the samples. Other investigations [102] reported that the impact strength and surface hardness of PMMA/ZrO_2_ nanocomposites was lower than that of control PMMA. Taking all these into account, it can be concluded that the addition of ZrO_2_ is not advantageous for odontological uses.

SiO_2_ NPs have also been added to PMMA to form nanocomposite materials for odontological uses [74,75,76,77]. These NPs enhanced the mechanical performance i.e., flexural modulus, surface hardness, dimensional stability, and thermal properties. However, the flexural strength was compromised due to the poor NP bonding with the PMMA matrix. On the other hand, the addition of SiO_2_ could have led to biocompatibility issues: the cytotoxic potential toward L929 and MRC5 cell lines was on the acceptable level for specimens with SiO_2_ loading lower than 2 wt%. Muhammad et al. [103] mixed PMMA with different loadings of amorphous SiO_2_ and TiO_2_ to yield nanocomposites for artificial teeth. In the presence of artificial saliva and under identical conditions, the wear resistance of the nanocomposites was better than that of commercial artificial teeth.

Addition of Al_2_O_3_ nanoparticles to PMMA resulted in good biocompatibility and improved thermal conductivity. Silane-treated Al_2_O_3_ noticeably improved the mechanical properties, specially the compressive and flexural strengths, as well as the wear resistance [69,70], while no significant effect on the water sorption or surface roughness of the resin were observed. Even though several works have shown promising results from using Al_2_O_3_, Rashahmadi et al. [104] used multi-criteria decision-making procedures, and reported that alumina is a less appropriate reinforcement for PMMA dentures compared to TiO_2_ and SiO_2_. They indicated that TiO_2_ nanoparticles are the most suitable among these three nanoparticles for odontological uses. In addition, the main drawback of alumina is that it induces the discoloration of the PMMA matrix.

**Figure 7:**
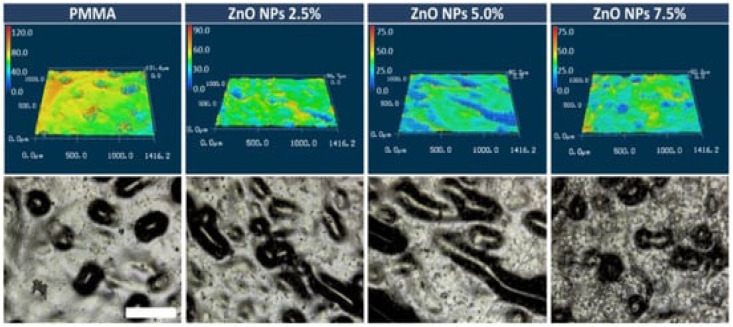
Surface topography of PMMA/ZnO nanocomposites: 3D laser microscope images (**top**) and optical laser-enhanced images (**bottom**). The scale bar (white) refers to 400 µm and it is applicable to all images. Reprinted from ref. [105], copyright 2018, MDPI.

The addition of ZnO NPs also increased the flexural and compressive strength of PMMA. The best performance was found at 7.5 wt% loading, ascribed to homogenous and random NP dispersion (Figure 7). Further, it was found that this composite had no cytotoxic effect on L929 cells [105] and showed enhanced antimicrobial activity. The reduced growth of microorganisms detected in PMMA/ZnO nanocomposites could be explained by their increased hydrophilicity and hardness.

**Figure 8:**
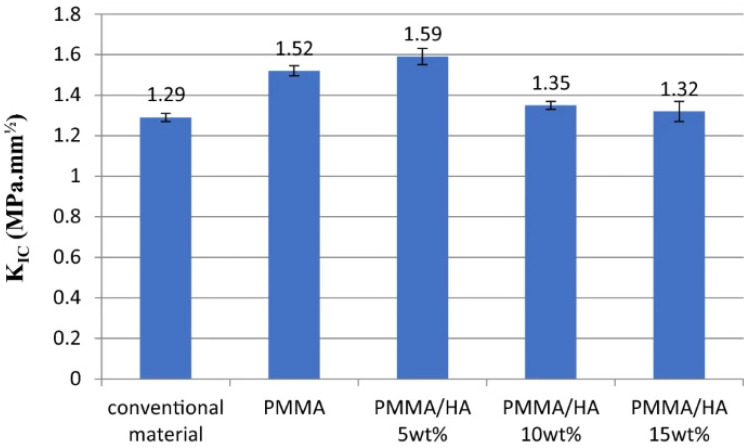
Effect of filler loading on the fracture toughness of PMMA/HA nanocomposite compared to the conventional material and neat PMMA. Reprinted from ref. [9], copyright 2020, MDPI.

Recently, several studies have been performed to investigate the effect of HA incorporation into PMMA as a bone substitute material. Zebarjad et al. [106] studied the mechanical properties of nano HA-reinforced PMMA using three-point bending, compressive, and wear tests and concluded that the addition of HA (up to 10 wt%) hardly changed the flexural properties of the matrix. Similar observations were reported by Wen et al. [107] who reported that the flexural strength and strain decreased in the presence of HA. Conversely, other studies [9,71] which treated HA with a silane coupling agent found that this nanofiller improved the mechanical properties of PMMA, including the elastic and flexural modulus that increased up to 26% and 27.3%, respectively, for the nanocomposite with 15 wt% HA loading. However, no significant differences were found in the tensile and flexural strength upon HA addition. Only a small increase of 1.6% and 3% in the tensile and flexural strength, respectively, was achieved at 5 wt% loading [9], while the surface hardness increased by about 8% with 15 wt% HA. The effect of HA loading on fracture toughness of PMMA/HA composites was also investigated (Figure 8) [9]. Neat PMMA exhibited poor fracture toughness, ascribed to the small plastic deformation of high molecular weight polymers. Upon the addition of HA, the fracture toughness increased and reached a maximum at a filler loading of 5 wt%, ascribed to the homogenous HA dispersion that inhibits crack propagation. However, a further increase in HA content decreased the fracture toughness due to NP agglomeration in the matrix. The agglomerates can act as obstructions to chain movement; they behave as stress concentration centers which initiate failure under stress.

### 4.3. PMMA Nanocomposites with Carbon-Based Nanofillers

CNTs have also been used to reinforce PMMA [13,21,108,109,110,111], attributed to their superior electrical and mechanical properties, low density, and resilience. Wang et al. [13] incorporated MWCNTs to PMMA and characterized the mechanical properties. The addition of MWCNT up to 1.0 wt% enhanced the flexural strength and resilience, while higher loadings decreased them due to CNT aggregation. The state of dispersion of the MWCNTs is a key factor that conditions the range of MWCNT reinforcement. Besides, according to Raman and dynamic loading experiments, the interfacial bonding between the MWCNTs and PMMA matrix was not strong enough. Also, the fatigue resistance of the matrix slightly decreased upon addition of MWCNTs. Similar observations were reported by Mahmood et al. [109], who found an increase in mechanical properties up to 1.5 wt% loading. Qasim and coworkers [110] added SWCNTs to light-cured PMMA, and they did not observe any significant effect on flexural strength. Kim et al. [111] used CNT loadings in the range of 0.25–2.0 wt%, and found a decrease in *C. albicans*, *S. aureus*, and *S. mutans* adhesion in the range of 35–95%. Nonetheless, despite the fact that some improvements in mechanical and antibacterial properties have been attained, there are many concerns about the use of CNTs for clinical purposes, the color in particular, which limits the use of PMMA/CNT nanocomposites in non-aesthetic areas.

**Figure 9:**
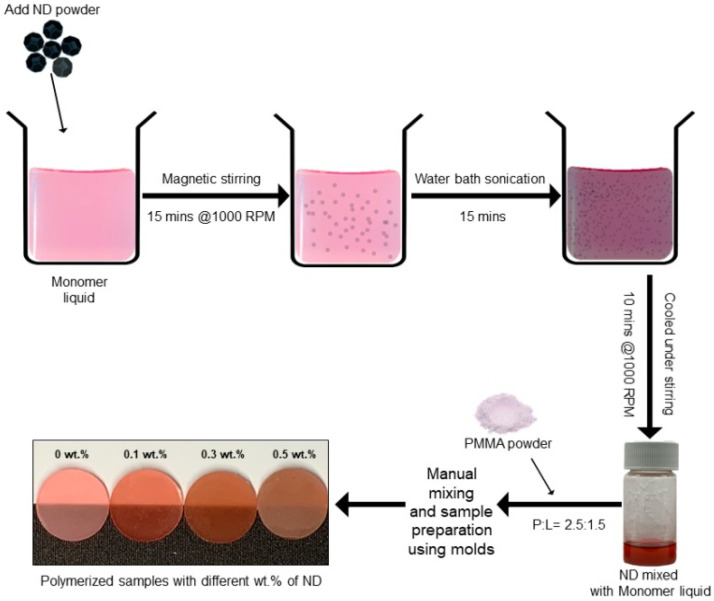
Representation of the preparation of PMMA/ND nanocomposites. Reprinted from ref. [112], copyright 2019, MDPI.

On the other hand, NDs have been incorporated into PMMA due to their excellent physical, chemical, and antibacterial properties, together with chemical stability, bioactivity, and biocompatibility [56,112,113]. The addition of 0.5 wt% ND to PMMA significantly increased its elastic modulus, flexural, and impact strength and decreased its surface roughness [56]. Mangal et al. prepared nanocomposites with 0.1, 0.3, and 0.5 wt.% ND loadings via addition of the ND powder in the liquid monomer, followed by simple stirring, baht sonication, and then UV-curing (Figure 9), and the nanocomposites were tested against neat PMMA and PMMA/ZrO. Flexural strength, elastic modulus, and surface hardness were evaluated, and all of them improved with increasing ND loading. Fungal adhesion and viability were studied using *C. albicans*, which were significantly reduced compared to the control. In addition, salivary biofilm formation was noticeably reduced compared to nanocomposites with ZrO. Hence, the incorporation of 0.1–0.5 wt.% ND improved mechanical properties and provided fungal resistance [112].

A summary of the improvements in PMMA properties upon addition of the different nanofillers is provided in Table 1.

## 5. Conclusions

Despite PMMA having been widely applied for odontological uses, it poses some limitations including discoloration, hydrolytic degradation, and poor resistance to impact and fracture. Therefore, a lot of work has been recently focused on the addition of different fillers to improve its properties to expand its use in dentistry. In this regard, nanofillers are highly suitable due to their low density, high specific surface area, and exceptional mechanical properties. Among the nanofillers that have been applied to reinforce PMMA are noble metals like AgNPs, AuNPs and PtNPs, metallic oxides and bioceramics such as TiO_2_, ZnO, ZrO_2_, Al_2_O_3_, and HA, as well as carbon-based nanomaterials like CNTs and NDs. These materials can provide improved mechanical and tribological characteristics, reduced water absorption, and long-term antifungal and antibacterial properties. Despite remarkable improvements in impact strength, flexural strength, wear resistance, and thermal conductivity, as well as the attainment of reduced water absorption and solubility, it remains challenging to improve one set of properties without compromising the rest. In particular, the addition of some nanoparticles compromises the aesthetics (color, translucency) or raises biocompatibility issues due to the leaching of degradation products in the oral cavity. Therefore, further investigation should be performed in this direction. The biocompatibility of the nanocomposites is sometimes questionable and requires additional examination. Further research should also focus on understanding the interactions between the nanofillers and PMMA resin at the molecular level. In addition, more studies should be carried out in vivo or under simulated oral conditions prior to the use of these novel nanocomposites in the field of dentistry.

## Figures and Tables

**Table 1:** Improvements in PMMA/nanofiller nanocomposites used for odontological applications.

Nanofiller (wt% or mg/L*)	Modification	Improvement	Ref.
AgNPs		Compressive strength, thermal conductivity	[10]
AgNPs	Reduced bacterial adhesion	Biocompatibility, antifungal activity	[86]
AgNPs		Reduced water absorption, compressive and flexural strength	[94]
AgNPs (3.0, 3.5)	Reduced surface roughness	Antibacterial activity	[95]
AgNPs (0.05)	Reduced bacterial adhesion	Antibacterial activity, compressive strength	[96]
PtNPs (50*)	Reduced bacterial adhesion	Flexural strength, thermal stability, melting point	[97]
PdNPs		Bending strength, Vickers hardness	[98]
AuNPs	Viscoelastic response	Thermal conductivity	[99]
TiO_2_ (1.0, 3.0)	Relaxation behavior	Hardness, modulus, creep-recovery, antibacterial activity	[78]
TiO_2_ (1.0)	Reduced bacterial adherence	Tensile strength, antibacterial activity	[100]
TiO_2_ (1.0, 5.0)		Microhardness, antifugal activity	[107]
ZrO_2_	Reduced translucency	Strength	[72]
ZrO_2_ (3.0, 5.0)		Fracture toughness, compressive and fatigue strength, thermal conductivity	[12]
SiO_2_ (2.0)	Weaker interfacial bonding	Flexural modulus, surface hardness, dimensional stability, thermal conductivity	[74]
Al_2_O_3_		Compressive and flexural strength, wear resistance	[69]
ZnO (7.5)	Hydrophilicity	Flexural and compressive strength, hardness, biocompatibility	[105]
HA (15)		Elastic and flexural modulus	[71]
HA (5.0)	Surface hardness	Fracture toughness	[9]
MWCNT (1.0)	Interfacial bonding	flexural strength and resilience	[13]
MWCNT	Color	Mechanical properties	[109]
MWCNT (0.25–2.0)	Color	Antibacterial activity	[111]
ND (0.5)		Elastic modulus, flexural and impact strength	[56]
ND (0.1–0.5)	Reduced salivary biofilm	Flexural strength, elastic modulus, surface hardness, antifungal activity	[112]

* concentration in mg/L.

## Data Availability

Not applicable.
